# Pancreatic Cancer and Benign Pancreatic Cystic Lesions: Differences in Cytokines, Growth Factors, and Immunological Markers Concentrations in Serum and Cystic Fluid

**DOI:** 10.3390/cancers17172783

**Published:** 2025-08-26

**Authors:** Ewa Grudzińska, Paweł Szmigiel, Karolina Majewska, Sławomir Mrowiec, Zenon P. Czuba

**Affiliations:** 1 Department of Gastrointestinal Surgery, Faculty of Medical Sciences in Katowice, Medical University of Silesia, 40-752 Katowice, Polandkarolina.majewska@sum.edu.pl (K.M.); smrowiec@sum.edu.pl (S.M.); 2 Department of Microbiology and Immunology, Faculty of Medical Sciences in Zabrze, Medical University of Silesia, 41-800 Zabrze, Poland; zczuba@sum.edu.pl

**Keywords:** pancreatic cancer, IPMN, pancreatic cystic tumors, biomarkers, cytokines, growth factors, pancreatoduodenectomy, angiogenesis

## Abstract

Background: Pancreatic cystic lesions may be benign and require observation or cancerous, with high mortality, requiring risky surgery. Diagnosis is often difficult, and the search for biomarkers to differentiate pancreatic cancer from other lesions is ongoing. Methods: 60 consecutive patients, operated on due to histopathologically confirmed pancreatic cancer or due to pancreatic cystic lesions, were analyzed. The concentrations of 16 immunological factors (sHER-2neu, sEGFR, sIL-6Ra, follistatin, FGF-basic, sVEGFR-2, PECAM-1, PDGF-AB BB, prolactin, G-CSF, HGF, sTIE-2, SCF, sVEGFR-1, osteopontin, and leptin) were assessed in both serum and cystic fluid and compared between the groups. Results: Lower PDGF-AB/BB and leptin concentrations in serum, as well as lower sTIE-2, osteopontin, and leptin levels, were associated with cancer. In cystic tumors, for some factors, significant differences between cancerous and benign lesions were found when the differences in cystic fluid and serum concentrations were compared. Conclusions: PDGF-AB/BB, leptin, sTIE-2, and osteopontin, as well as the comparison of serum/cystic fluid concentrations of immunological factors, might be useful for pancreatic cystic tumor diagnosis. However, this requires confirmation in a larger study.

## Simple Summary

Pancreatic cystic tumors require risky surgery when malignancy is suspected. To avoid unnecessary interventions and make a precise diagnosis easier, the search for biomarkers of pancreatic cancer is ongoing. To our knowledge, there are no studies on immunological factors assessed in the cystic fluid from removed tumors. We analyzed a panel of 16 immunological factors in a group of 60 patients undergoing pancreatic surgery due to pancreatic cancer or benign pancreatic cystic lesions. We found an association between cancer and lower serum concentrations of PDGF-AB/BB and leptin. Also, lower concentrations of sTIE-2, osteopontin, and leptin in the cystic fluid were associated with malignant lesions. In patients with cystic tumors, the concentrations of some markers differed between serum and cystic fluid. The described factors present possible future biomarkers for pancreatic lesion diagnostics, and further research in larger groups of patients is required.

## 1. Introduction

Pancreatic ductal adenocarcinoma (PDAC) is one of the most prevalent causes of cancer-related death, with a very low 5-year survival rate estimated at 11% [1]. One of the causes is the late detection of the cancer, due to its asymptomatic course until the advanced stage. When diagnosed early, it allows for a 5-year survival of 80%, which makes the search for new markers an important issue [1,2]. Adenocarcinomas are the vast majority of solid pancreatic tumors [3]. However, among cystic pancreatic lesions, the diagnosis may be more challenging, and the decision on whether to perform risky surgical treatment is more difficult. While some cystic lesions are benign and mostly require surveillance, others have a high risk of malignant transformation and should be resected (Table 1).

Depending on pancreatic tumor localization, either pancreatoduodenectomy (PD) or distal pancreatectomy (DP) with splenectomy is indicated. For multifocal lesions or when pancreatic texture is assessed as extremely risky for anastomosis, total pancreatectomy (TP) may be necessary [6,7]. The complication rate for PD can reach 50% [8]. For DP and TP, the severe morbidity rate is up to 32% [9,10].

Because of the high mortality in advanced pancreatic cancer, diagnostic difficulties in cystic tumors, and the serious complications associated with pancreatic surgery, there is a significant need to find additional diagnostic tools that could help in the early detection and differentiation of pancreatic lesions. Apart from imaging methods (abdominal ultrasound, computed tomography, endoscopic ultrasound-EUS [11,12]), the search for markers that may improve diagnostics is ongoing. One commonly used marker, serum carbohydrate antigen (CA 19-9), lacks sufficient specificity for screening purposes [13]. It is assessed both in the serum and in the cystic fluid. Tumor biopsy and acquisition of the cystic fluid during EUS, via fine needle aspiration (FNA), is a widely accepted diagnostic method [11]. Finding additional PDAC markers in the cystic fluid could improve early PDAC diagnosis.

Immunological factors such as cytokines, chemokines, growth factors, and angiogenesis factors have been studied as potential diagnostic markers in PDAC; however, none have yet been qualified for use in clinical conditions due to the scarcity of such studies and relatively small examined groups [14,15,16]. There is a need to distinguish the potentially promising factors and to validate them in larger, possibly prospective, multicenter studies.

Our study aimed to analyze a panel of 16 immunological factors, known to take part in angiogenesis, cell growth, and migration, to establish whether there are any significant differences in their concentrations in serum between patients with cancer and patients with benign cystic lesions. We also analyzed the cystic fluid concentrations, looking for differences between cancerous and benign cysts. In the cystic lesion group, we also assessed the differences in the concentrations between the cystic fluid and serum in cancer and non-cancer subgroups. Our goal was to identify potentially useful candidates for biomarkers to differentiate between benign and cancerous lesions for future analyses in larger patient groups.

## 2. Materials and Methods

We analyzed 60 consecutive patients, operated on due to histopathologically confirmed pancreatic cancer or due to pancreatic cystic lesions. The exclusion criteria were as follows: pregnancy, inflammatory diseases, current smoking, current alcohol use disorder, anti-inflammatory drug intake in the last two weeks. Eighteen were male (30%) and fourty-two were female (70%). The patients were afterwards divided into two groups according to the postoperative histopathology results: 40 patients (67%) in the cancer group (both solid and cystic lesions), and 20 patients (33%) in the non-cancer group (with cystic lesions).

**Figure 1:**
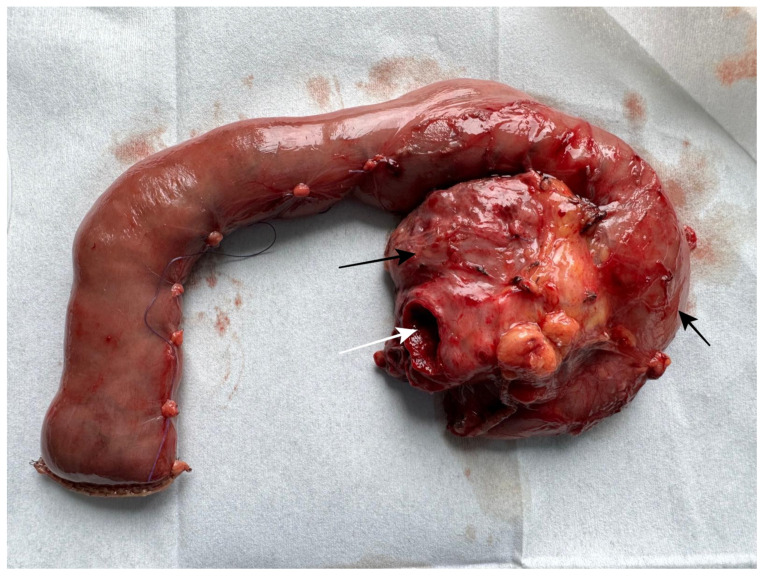
Postoperative specimen after pancreatoduodenectomy, dorsal view. Long black arrow—IPMN-MD tumor; short black arrow—duodenum; white arrow—widened main pancreatic duct.

Serum samples were obtained preoperatively from 59 patients (98.33%)—40/40 (100%) in the cancer group and 19/20 (95%) in the non-cancer group. The serum was centrifuged, and the supernatant was frozen at −80 °C for further analysis. Cystic fluid samples were obtained from 32 patients (53.33%)—16/40 (40%) in the cancer group and 16/20 (80%) in the non-cancer group, intraoperatively, directly after the tumor removal (Figure 1), via aspiration with a needle (1.2 × 40 mm; 18 G) and syringe. The volume of the acquired fluid was 0.5–2.0 mL.

The fluid was centrifuged, and the supernatant was frozen at −80 °C for further analysis. After all samples were obtained, we analyzed the demographic data, the incidence of preoperative diabetes, the preoperative serum glucose concentrations, the white blood cell count (WBC), the lymphocyte count, the C-reactive protein (CRP) concentrations, the postoperative histopathology results, and the staging and grading of the cancers. The serum and cystic fluid were thawed, and the concentrations of 16 factors (sHER-2neu, soluble epidermal growth factor—sEGFR, soluble interleukin-6 receptor alpha—sIL-6Ra, follistatin, fibroblast growth factor—FGF-basic, soluble vascular endothelial growth factor receptor-2—sVEGFR-2, platelet endothelial cell adhesion molecule-1—PECAM-1, platelet-derived growth factor—AB/BB—PDGF-AB/BB, prolactin, granulocyte colony—stimulating factor—G-CSF, hepatocyte growth factor—HGF, soluble TIE-2—sTIE-2, stem cell factor—SCF, soluble vascular endothelial growth factor-1—sVEGFR-1, osteopontin, and leptin) were assessed using Bio-Plex Pro™ Assays (Bio Rad Laboratories, Hercules, CA USA) according to the manufacturer’s instructions. The sample volume required for the analysis was 50 μL, and all samples were sufficient. In the cancer group, in four patients, the acquired fluid was too viscous to perform the Bio-Plex Pro™ Assay, even after the fluid was diluted with saline; in these patients, only serum samples were included.

Statistical analysis was conducted using IBM SPSS Statistics 30.0. Qualitative variables were presented as absolute values with their percentages. Quantitative variables were expressed as means with standard deviations (SDs) or medians with interquartile ranges (IQRs). The Shapiro–Wilk test was used to verify the distribution of the quantitative variables. Levene’s test was applied to verify the homogeneity of variance. Between-group differences were tested using the chi-square test or Fisher’s exact test for qualitative variables and the Student *t*-test or the Mann–Whitney test for quantitative variables, depending on the distribution of the variables. Univariate logistic regression analysis was used to assess the relationship between the serum or cystic fluid parameters and the presence of cancer. The differences in fluid and serum parameters were analyzed with a paired Student *t*-test for variables with normal distribution or Wilcoxon signed-rank test for non-normally distributed data. A *p*-value of <0.05 was considered statistically significant.

The study was conducted in accordance with the Declaration of Helsinki and approved by the Institutional Ethics Committee of Medical University of Silesia (PCN/CBN/0022/KB1/119/I/21/22, 18 January 2022). Informed consent was obtained from all subjects involved in this study.

## 3. Results

In the analyzed group, there were 42 women (70%) and 18 men (30%). The patients in the cancer group were significantly older (mean age 67 years, compared to 51 in the non-cancer group, *p* < 0.001), had significantly higher serum glucose levels (105 vs. 92.8, *p* < 0.002), and more often had preoperative diabetes (13 vs. 1, *p* < 0.023). There were no statistically significant differences in CRP, WBC, or lymphocyte count. Table 2 presents the general characteristics of the examined groups.

A total of 40 patients were diagnosed with pancreatic adenocarcinoma (60%), of which 25 had solid tumors (4 of these patients underwent bypass surgery without resection but had PDAC confirmed in preoperative biopsy, and 1 patient had PDAC metastases diagnosed in the resected liver lesions), and 15 had cystic tumors: 12 had IPMN with adenocarcinoma, and 3 had MCN with adenocarcinoma.

A total of 20 patients had benign cystic tumors: IPMN (*N* = 1, 1.67%), MCN without high-grade dysplasia (*N* = 8, 13.33%), SCN (*N* = 6, 10%), SPN (*N* = 2, 3.33%), MCN with SCN (*N* = 1; 1.67%), lymphoepithelial cyst—LEC (*N* = 1, 1.67%), cystic neuroendocrine tumor—NET (*N* = 1, 1.67%).

**Figure 2:**
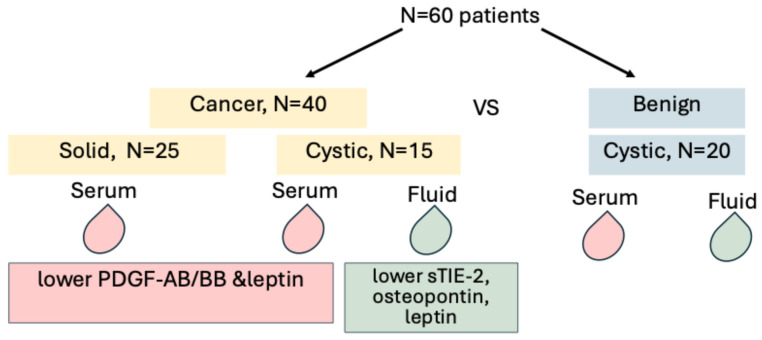
Graphical representation of the major findings. Of 60 patients, 40 had cancer (25 solid tumors and 15 cystic tumors), and 20 patients had benign cystic tumors. Lower PDGF-AB/BB and leptin were associated with cancer, as well as lower sTIE-2, osteopontin, and leptin in the cystic fluid.

In the serum samples, lower PDGF-AB/BB and leptin concentrations were associated with PDAC occurrence (0.2 ng/mL vs. 0.4 ng/mL, OR = 0.449, 95%CI = 0.221–0.913; *p* = 0.027, and 1 ng/mL vs. 2.1 ng/mL, OR = 0.654, 95%CI = 0.471–0.907; *p* = 0.011, respectively). In the cystic fluid, lower sTIE-2, osteopontin, and leptin concentrations were related to PDAC diagnosis (1 ng/mL vs. 0.9 ng/mL, OR = 0.231, 95%CI = 0.054–0.990; *p* = 0.048; 13.24 vs. 156.2 ng/mL, OR = 0.960, 95%CI = 0.923–0.998; *p* = 0.039; 0.2 vs. 2.7, OR = 0.158, 95%CI = 0.037–0.677; *p* = 0.013, respectively). Other analyzed factors were not associated with cancer occurrence (Figure 2, Table 3 and Table 4).

For the cystic lesions, we compared the differences in concentrations of the analyzed factors between serum and cystic fluid for the cancer and non-cancer groups. In the cancer group only, differences in marker concentrations between serum and cystic fluid were observed for sIL-6Ra, sVEGFR-2, PECAM-1, and SCF. These factors had lower concentrations in the cystic fluid than in serum (1.3 vs. 7.8 ng/mL; *p* < 0.001, 0.3 vs. 0.7 ng/mL; *p* = 0.039, 2.8 vs. 5.5 ng/mL; *p* = 0.011, and 0.1 vs. 0.2 ng/mL, *p* = 0.037, respectively).

In the non-cancer group only, sHER-2neu, follistatin, and leptin were significantly higher in the cystic fluid than in the serum (5.3 vs. 3.2 ng/mL; *p* = 0.026,0.7 vs. 0.3 ng/mL, *p* = 0.005, and 2.7 vs. 2.1 ng/mL; *p* = 0.026, respectively), and PDGF-AB/BB was significantly higher in the serum (0.1 vs. 0.4 ng/mL; *p* = 0.039).

**Figure 3:**
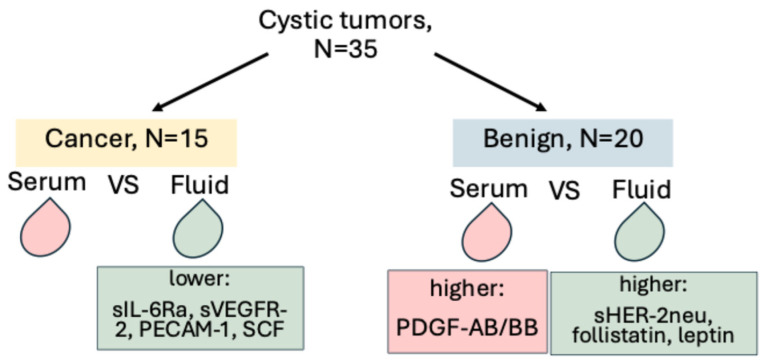
Graphical representation of the major findings. Of 35 patients with cystic tumors, 15 had cancer, and 20 had benign cystic tumors. When the serum vs. fluid concentrations in these patients were compared, in the cancer group, lower cystic fluid concentrations were found to be significant for sIL-6Ra, sVEGFR-2, PECAM-1, and SCF. Higher fluid concentrations of sHER-2neu, follistatin, and leptin, and higher serum concentrations of PDGF-AB/BB were found to be significant in the benign tumors.

FGF-basic, HGF, sVEGFR-1, and osteopontin were significantly higher in the cystic fluid in both groups. Prolactin and sTIE-2 were significantly lower in the cystic fluid in both groups (Figure 3, Table 5).

## 4. Discussion

The diagnostic process of cystic pancreatic lesions usually consists of imaging tests (contrast-enhanced computed tomography and/or magnetic resonance imaging/MRI/and/or cholangio-MRI), EUS with or without biopsy [12], and markers such as carcinoembryonic antigen (CEA) and CA 19-9, checked in the serum and cystic fluid obtained during EUS. The preoperative diagnosis is incorrect in 20–30% of cases in cystic tumors of the pancreas [17]. When cystic fluid markers are obtained, the differentiation between mucinous and non-mucinous cysts has an estimated diagnostic accuracy of 80%, still leaving 20% of the lesions with uncertain diagnosis [17].

PDAC has a very high mortality, and pancreatic resections have a very high risk of severe complications. Therefore, the decision of the treatment requires caution and additional markers enabling accurate and, if possible, non-invasive diagnosis, which are under investigation. Promising factors include DNA- and microRNA-based biomarkers, proteins (with some promising results on interleukin-1 (IL-1), VEGF-A, and tumor growth factor alpha—TGF-α); also, other cytokines, chemokines, and angiogenetic factors have been studied [17]. Some studies have shown better diagnostic performance when markers were combined, e.g., CA19-9 with IP-10, IL-6, and IL-8 performed better than CA19-9 alone [18]. In an analysis of cystic fluid, mucin, CEA levels, and VEGF-A were proven to have efficacy in discriminating premalignant and malignant lesions from benign lesions [13].

In our study, in a panel of 16 immunological factors, lower serum concentrations of PDGF-AB/BB and leptin were associated with cancer, and in the cystic fluid, cancerous tumors were associated with lower concentrations of sTIE-2, osteopontin, and leptin.

### 4.1. PDGF AB/BB

PDGF is a mitogen and chemoattractant, taking part in the development of several cancers [19]. It has different isoforms, of which the PDGF-AA, PDGF-AB, and PDGF-BB dimers are activated intracellularly before secretion. PDGF binds to two types of receptors—PDGFRα and PDGFRβ [19]. It has been shown to increase pancreatic stellate cell (PSC) activation and the production of extracellular matrix proteins [20,21]. An autonomous autocrine-signaling pathway for PDGF-A was described, mediating growth, invasion, metastasis, and chemotherapy resistance in pancreatic cancer [22]. PDGF was shown to promote cell proliferation and enhance the aggressiveness of pancreatic cancer cells in vitro [23,24]. PDGFRβ was proven essential for the sustained expression of mutant p53, which promotes metastasis in murine models of pancreatic cancer. Blockade of this receptor prevented cancer invasion in vitro and metastasis in vivo, and its high expression correlated with worse patient survival [25]. In a study by Sakamoto et al., however, the PDGF-BB plasma concentrations were the same as in controls [26].

On the other hand, in a murine pancreatic cancer model, the authors found that in tumors overexpressing PDGF-BB, tumor growth was suppressed, and treatment of these tumors with PDGFR inhibitor (imatinib mesylate) resulted in increased growth and decreased total pericyte content [27]. Also, available studies found lower PDGF-AA serum levels in PDAC patients compared to benign pancreatic diseases, and cancer patients with higher circulating PDGF-BB concentrations had a better prognosis [14,16,28]. In a study assessing postoperative changes in immunological factors in PDAC, an increase in PDGF was associated with a better prognosis [16].

As a diagnostic tool, PDGF paired with CA19-9, IP-10, and IL-6 gave better results in discriminating PDAC and benign diseases in jaundiced patients [18].

In our study, malignant lesions showed lower PDGF AB/BB concentrations in the serum, which is in line with some of the available studies. In the group with benign cystic lesions, there was significantly lower concentrations in the cystic fluid when compared to the serum of the patients.

The available data show that PDGF has multiple functions in pancreatic cancer, and it is a possible biomarker as well as a treatment target.

### 4.2. Osteopontin

OPN is a protein associated with the extracellular matrix, extensively studied as a cancer biomarker. It was found to have a role in pancreatic cancer, as well as in cancers of the breast, ovaries, prostate, esophagus, liver, and bone [29]. It is expressed and secreted in activated PSCs, and it is involved in the PSC-induced epithelial–mesenchymal transition. It induces malignant phenotypes of PDAC cells [30]. Previously, it was reported to be upregulated in serum in PDAC, with a sensitivity and specificity of 80% and 97% [31], and when paired with CA19-9, it improved the diagnostic performance [13,32]. In a study of PDAC tissue, OPN mRNA was preferentially expressed in samples of patients with shorter survival after surgery [33]. It showed potential in differentiating PDAC from chronic pancreatitis; also, stage IV PDAC had higher OPN levels than stage III, but no differences were found between stages III and II [34].

On the other hand, outcomes of PDAC tissue analyses are contradictory. In one study, OPN was significantly upregulated in PDAC tissues and was associated with worse clinical outcomes [30]. In another study, a human PDAC tissue microarray was performed on 57 cancer samples, and neither the quantity nor the intensity of OPN staining correlated with PDAC grade, stage, T, or N status [35]. It is worth noting that OPN gene expression is elevated in pancreatic tissue in diabetes, and elevated glucose stimulates OPN secretion [36,37]. In our study, cancer patients had diabetes and elevated glucose in serum significantly more often; however, OPN did not differ significantly between groups in the serum. Lower concentrations in cystic fluid were associated with cancer, and when we compared the difference in serum/cystic fluid concentrations, both the cancer group and the non-cancer group showed significantly higher OPN concentrations in the patients’ cystic fluid than in the serum. The exact role of osteopontin in cystic tumors remains unclear, and we have not found any studies assessing OPN concentrations in pancreatic cystic tumor fluid.

### 4.3. Leptin

Leptin is a hormone, an adipokine produced by fat tissue, with higher concentrations in people with obesity and diabetes. Obesity is recognized as a PDAC risk factor; therefore, leptin is expected to have a role in this cancer’s development. In a murine model, animals with induced obesity PDAC grew larger, and serum leptin was higher [38]. The overexpression of leptin in mice promoted tumor growth and lymph node metastasis [39]. In vitro, leptin stimulation increases cellular migration, and in vivo, in murine models, depletion of the leptin receptor reduced orthotopic tumor growth in obese mice [40]. Leptin’s receptor, Ob-Rb was found in PDAC cell lines, but in vitro, leptin did not affect the proliferation of human PDAC cells [39]. In a large cohort study, serum leptin was not found to be generally associated with pancreatic cancer; however, a relationship was found in a long follow-up of over 10 years [41].

In our study, lower leptin concentrations were associated with cancer occurrence, both in serum and in the cystic fluid, even though BMI was not significantly different between groups. In the cancer group, diabetes was more prevalent, which would imply that higher leptin levels should be expected.

These results partly agree with an analysis of serum leptin in 235 IPMN patients, where leptin was significantly lower in more advanced IPMNs than in low-grade tumors, but, in females only [42]. In a study on pancreatic neuroendocrine tumors, the examined group had the same leptin levels in the serum as controls, with no BMI differences between groups [43]. In a large study with Mendelian randomization, leptin and its receptor were unrelated to pancreatic cancer risk [44]. Also, in a study by Man et al., the serum levels of leptin in PDAC patients were similar to controls, irrespective of the presence of diabetes. Obese and overweight patients had a lower survival rate, but they also also lower leptin levels [45]. Sakamoto et al. did not find significant differences in leptin levels between PDAC and control groups with pancreatitis and benign diseases [26]. In our comparison of the differences in the leptin concentrations in the serum/cystic fluid, leptin was significantly higher in the cystic fluid only in the group with benign cystic lesions. These data suggest that higher leptin concentrations may be a negative cancer biomarker.

### 4.4. sTIE-2

sTIE-2 is a receptor for angiopoietin-1 (Ang-1) and its antagonist, angiopoietin-2 (Ang-2). It is expressed in endothelial cells, hematopoietic stem cells, and monocytes. Tie2^+^ macrophages are known to be pro-angiogenic, pro-metastatic, and immunosuppressive in the tumor microenvironment [46]. Ang-1 supports vascular integrity, while binding with Ang-2 causes vascular instability and promotes a response to mitogens. In tumors, the balance is shifted towards Ang-2, which in the presence of VEGF is an inducer of angiogenesis, increasing neovascularization [47]. In patients with pancreatic neuroendocrine tumors, circulating Ang-2 was significantly elevated compared with healthy controls, and it correlated with metastatic disease and shorter survival. The authors checked the Ang-2/sTie-2 ratio, but it did not improve the correlation to clinicopathologic parameters [48]. One study assessed the reaction of PSCs to hypoxia because hypoxic conditions were found in pancreatic tumors. Hypoxia induced migration and VEGF production in PSCs, which also expressed the Tie-2 receptor [21]. Tie-2 was also found to play a role in angiogenesis inhibition in PDAC by foretinib, which blocks Tie-2 activation [49]. In another analysis, Tie-2 was not differentially expressed in PDAC or pancreatic neuroendocrine tumors when compared to normal pancreatic tissue; however, overexpression of Ang-2 gene and its gene products was found, indicating a role of Ang/Tie-2 in pancreatic tumors [50].

In our study, lower sTie-2 in cystic fluid was associated with the cancerous lesions, and no difference was found in the serum between groups. It was lower in the cystic fluid than in the serum, both in patients with cancerous and benign cysts. The role of this receptor in PDAC and cystic tumors requires further investigation.

### 4.5. Other Differences Between Serum and Cystic Fluid

Apart from the above, in our analysis of the differences in the concentrations of the immunological factors in serum and cystic fluid, between the cancer and non-cancer groups of patients with cystic tumors, only the cancer group showed significantly lower sIL-6Ra, sVEGFR-2, PECAM-1, and SCF in the cystic fluid than in the serum.

Only in the non-cancer group, sHER-2neu and follistatin were significantly higher in the cystic fluid. Both groups showed significantly higher FGF-basic, HGF, and sVEGFR-1 in the cystic fluid than in the serum, as well as significantly lower prolactin in the cystic fluid than in the serum.

In a study of the plasma levels of angiogenesis-related factors, VEGF, follistatin, HGF, or PECAM-1 were not significantly different in PDAC patients compared with control groups [26]. To our knowledge, there are no available studies comparing serum and cystic fluid immunological biomarker concentrations in pancreatic tumors. The serum/fluid differences found in our study might prove useful for cystic lesion differentiation.

The small size of the examined groups are a major weakness of our study. The decision to include only cystic fluid samples from surgically removed tumors reduced the number of patients eligible for this study. However, for scientific purposes, we needed to obtain a full histopathology result from the cystic tumors, as EUS biopsies have a relatively high false-negative rate [51]. Due to the exploratory nature and the limited sample size, correction for multiple comparisons was not applied. Also, no formal a priori power calculation was performed, as the sample size was determined by the availability of eligible patients during the study period. Moreover, given the sample size, multivariable adjustment was not feasible. Therefore, the findings should be interpreted with caution and confirmed in larger cohorts with multivariate models.

It must be noted that immunological markers are prone to several factors that may alter their concentrations. We applied exclusion criteria to limit this bias; however, we did not exclude diabetic patients from this study, despite the known impact of diabetes on immunological status. We made this decision because diabetes is known to be more prevalent in patients with pancreatic tumors, and excluding them would severely further reduce the number of patients eligible for the analysis. It must be noted, however, that this might impact the results. A large, possibly multicenter study would be needed to recruit a larger examined group, excluding this interfering factor.

Another limitation of our work is the single-center setting and the retrospective character of the study. To confirm the possible clinical usefulness of the markers, a study on a larger group, possibly in a multicenter setting, is needed. For now, the limitations of the study do not allow us to recommend any of the presented relevant markers for use in a clinical setting; however, it provides a possible direction for future studies. We also showed, to our knowledge, for the first time, that the comparison of the examined markers’ concentrations in the cyst fluid and serum might be a useful direction in the search for better diagnostics. Once relevant markers are confirmed in the cystic fluid, samples can be acquired via EUS-FNA.

## 5. Conclusions

In the search for biomarkers that may prove useful in the differential diagnosis of benign cystic pancreatic tumors and PDAC, we have found that lower PDGF-AB/BB and leptin concentrations in serum were associated with cancer, and lower concentrations of sTIE-2, osteopontin, and leptin in the cystic fluid may be associated with cancerous tumors. Also, in cystic tumors, the difference in concentrations between the cystic fluid and serum seems to be a promising way to differentiate cancerous and benign lesions. However, further studies with larger examined groups are required.

## Figures and Tables

**Table 1:** The most common pancreatic cystic lesions and recommended treatment. Abbreviations: MCN—mucinous cystic neoplasm, SPN—solid papillary neoplasm, SCN—serous cystic neoplasm, IPMN-MD/mix type/BD—intraductal papillary mucinous neoplasm main duct/mix type/branch duct [4,5].

Pancreatic Cystic Lesion Type	Recommended Treatment
MCN	Surgery
SPN	Surgery
SCN	Observation
IPMN-MD	Surgery
IPMN mix type	Surgery
IPMN-BD	Observation/surgery when malignant features are present

**Table 2:** General characteristics of the examined groups. BMI—body mass index, ASA class—American Society of Anesthesiologists physical status class, WBC—white blood cell count, CRP—C-reactive protein.

Variable	Total (*n* = 60)	Cancer Group (*n* = 40, 66.67%)	Non-Cancer Group (*n* = 20, 33.33%)	*p*
**Female sex**	42 (70%)	26 (65%)	16 (80%)	0.232
**Age (years)**	65 (24–79, IQR = 18)	67 (43–79, IQR = 10)	51 (24–75, IQR = 24)	**<0.001**
**BMI (kg/m^2^)**	25.2 (17.7–39.1, SD = 4.12)	24.9 (17.7–33.4, SD = 3.65)	25.9 (17.9–39.1, SD = 4.97)	0.393
**ASA class** **II** **III** **IV**	24 (40%) 35 (58.3%) 1 (1.7%)	12 (30%) 27 (67.5%) 1 (2.5%)	12 (60%) 8 (40%) 0	0.073
**Preoperative diabetes**	14 (23.3%)	13 (32.5%)	1 (5%)	**0.023**
**WBC [10^3^/mm^3^]** **Lymphocytes** **[10^3^/mm^3^]** **CRP [mg/L]** **Serum glucose [mg/dL]**	6.4 (2.8–9.8, SD = 1.53) 1.6 (0.7–2.7, SD = 0.44) 1.2 (0.6–25.8, IQR = 1.9) 97.5 (77.5–157, IQR = 22.3)	6.3 (3.6–9.8, SD = 1.46) 1.6 (0.7–2.7, SD = 0.5) 1.4 (0.6–25.8, IQR = 1.7) 105 (78.5–157, IQR = 40.2)	6.7 (2.8–9.5, SD = 1.66) 1.7 (1.1–2.2, SD = 0.33) 0.8 (0.6–12.1, IQR = 2.6) 92.8 (77.5–110, IQR = 11.3)	0.333 0.550 0.110 **0.002**

**Table 3:** Association of serum concentrations of the assessed factors with PDAC occurrence.

Variable	Total (*n* = 59)	Cancer Group (*n* = 40, 67.8%)	Non-Cancer Group (*n* = 19, 32.2%)	*p*	OR (95%CI)
**sHER-2 neu [ng/mL]**	2.8 (0.08–8.66, IQR = 2.54)	2.7 (0.08–8.66, IQR = 2.65)	3.2 (1–6.7, IQR = 2.54)	0.095	0.762 (0.554–1.048)
**sEGFR** **[ng/mL]**	6.3 (0.32–26.2, IQR = 6.93)	6 (0.32–26.21, IQR = 6.43)	8.1 (0.61–20.94, IQR = 7.31)	0.972	0.998 (0.915–1.090)
**sIL-6Ra** **[ng/mL]**	8.2 (2.1–25.42, IQR = 5.41)	7.8 (2.1–22.12, IQR = 4.32)	10.1 (3.66–25.42, IQR = 5.85)	0.279	0.937 (0.833–1.054)
**Follistatin [ng/mL]**	0.2 (0.001–0.69, IQR = 0.26)	0.2 (0.001–0.67, IQR = 0.29)	0.3 (0.09–0.69, IQR = 0.25)	0.076	0.056 (0.002–1.356)
**FGF-basic** **[pg/mL]**	108.3 (21.71–256.31, SD = 72.32)	105 (22–254, SD = 55.7)	114 (29–256, SD = 53.5)	0.547	0.997 (0.987–1.007)
**sVEGFR-2 [ng/mL]**	0.8 (0.31–1.61, IQR = 0.51)	0.7 (0.31–1.61, IQR = 0.33)	1 (0.4–1.54, IQR = 0.38)	0.051	0.166 (0.027–1.008)
**PECAM-1** **[ng/mL]**	5.5 (2–8.84, SD = 2.1)	5.4 (2–8.84, SD = 1.56)	5.6 (3.39–8.51, SD = 1.4)	0.587	0.903 (0.624–1.306)
**PDGF-AB/BB** **[ng/mL]**	0.3 (0.001–4.54, IQR = 0.58)	0.2 (0.001–1.79, IQR = 0.57)	0.4 (0.013–4.54, IQR = 1.84)	**0.027**	**0.449** **(0.221**–**0.913)**
**Prolactin** **[ng/mL]**	7 (0.01–143.8, IQR = 24.83)	5.7 (0.01–108.67, IQR = 21.06)	8.5 (0.01–143.8, IQR = 59.42)	0.110	0.985 (0.967–1.003)
**G-CSF** **H-[pg/mL]**	101 (3.37–249.64, IQR = 43.29)	97 (3–250, IQR = 76.5)	111 (75–175, IQR = 33.8)	0.188	0.992 (0.980–1.004)
**HGF** **[pg/mL]**	1021.5 (210.9–2235.83, IQR = 813.93)	981 (211–2236, IQR = 834.9)	1104 (300–1962, IQR = 728.8)	0.774	1.000 (0.999–1.001)
**sTIE-2 [ng/mL]**	4.1 (0.16–17.01, IQR = 5.91)	3.7 (0.16–17.01, IQR = 5.96)	5.6 (0.42–11.43, IQR = 5.65)	0.547	0.957 (0.829–1.105)
**SCF** **[pg/mL]**	159 (19.3–379.88, SD = 72.41)	166 (19–380, SD = 71.9)	145 (53–288, SD = 73.3)	0.303	1.004 (0.996–1.012)
**sVEGFR-1** **[pg/mL]**	0.6 (0.33–4.98, IQR = 2.75)	0.5 (0.3–5, IQR = 3.08)	0.6 (0.4–4.4, IQR = 1.41)	0.447	1.161 (0.790–1.707)
**Osteopontin [ng/mL]**	2.9 (0.06–17.85, IQR = 3.88)	3 (0.1–17.85, IQR = 5.48)	2.6 (0.06–6.1, IQR = 3.31)	0.131	1.165 (0.956–1.421)
**Leptin [ng/mL]**	1.2 (0.004–17.03, IQR = 1.85)	1 (0.004–4.6, IQR = 1.17)	2.1 (0.05–17.03, IQR = 5.76)	**0.011**	**0.654** **(0.471**–**0.907)**

**Table 4:** Association of cystic fluid concentrations of the assessed factors and PDAC occurrence.

Variable	Total (*n* = 32)	Cancer Group (*n* = 16, 50%)	Non-Cancer Group (*n* = 16, 50%)	*p*	OR (95%CI)
**sHER-2 neu [ng/mL]**	3.3 (0.01–27.62, IQR = 5.29)	2.7 (0.01–13.63, IQR = 3.99)	5.3 (0.44–27.62, IQR = 12.87)	0.110	0.874 (0.741–1.031)
**sEGFR [ng/mL]**	4.9 (0.06–33.67, IQR = 5.43)	2.6 (0.06–18.1, IQR = 4.71)	5.6 (0.74–33.67, IQR = 10.05)	0.102	0.871 (0.737–1.028)
**sIL-6Ra [ng/mL]**	2.6 (0.01–27.47, IQR = 5.23)	1.3 (0.01–6.36, IQR = 2.45)	4.4 (0.28–27.47, IQR = 15.09)	0.067	0.757 (0.562–1.020)
**Follistatin [pg/mL]**	398.4 (40.63–1820.63, IQR = 510.7)	287 (40.63–1820.63, IQR = 495.9)	683 (60–1554, IQR = 458)	0.308	0.999 (0.997–1.001)
**FGF-basic [pg/mL]**	210.8 (49.77–909.89, IQR = 222.6)	211 (49.77–909.89, IQR = 270.2)	217 (95–893, IQR = 197.2)	0.954	1.000 (0.997–1.003)
**sVEGFR-2 [ng/mL]**	0.5 (0.01–4.98, IQR = 0.98)	0.3 (0.01–1.2, IQR = 0.7)	0.7 (0.1–4.98, IQR = 1.34)	0.060	0.242 (0.055–1.061)
**PECAM-1 [ng/mL]**	3.88 (0.15–21.96, IQR = 5.12)	2.8 (0.15–7.58, IQR = 4.33)	4.5 (0.68–21.96, IQR = 8.23)	0.050	0.800 (0.641–1.000)
**PDGF-AB/BB [pg/mL]**	149.2 (10.75–1853.95, IQR = 198.69)	175 (10.75–1853.95, IQR = 506.3)	114 (12–252, IQR = 177.1)	0.086	1.005 (0.999–1.010)
**Prolactin [ng/mL]**	1 (0.06–11.12, IQR = 1.64)	0.7 (0.06–3.41, IQR = 1.37)	1.2 (0.14–11.12, IQR = 3.22)	0.123	0.613 (0.329–1.142)
**G-CSF [pg/mL]**	121.4 (12.59–742.5, IQR = 195.8)	121 (12.59–370, IQR = 142.7)	142 (18–742.5, IQR = 204)	0.202	0.996 (0.990–1.002)
**HGF [ng/mL]**	7.8 (0.002–100.85, IQR = 35.45)	3.29 (0.04–43.87, IQR = 8.53)	16.6 (0.002–100.85, IQR = 78.32)	0.056	0.949 (0.900–1.001)
**sTIE-2** **[ng/mL]**	1 (0.14–9.54, IQR = 1.1)	0.9 (0.14–1.53, IQR = 0.74)	1.6 (0.23–9.54, IQR = 3.05)	**0.048**	0.231 (0.054–0.990)
**SCF [pg/mL]**	108 (2.57–602.1, IQR = 142.27)	77 (2.57–257, IQR = 131.2)	133 (15–602.1, IQR = 267)	0.068	0.993 (0.986–1.000)
**sVEGFR-1 [ng/mL]**	1.7 (0.001–20.31, IQR = 5.12)	0.6 (0.001–7.42, IQR = 2.18)	3 (0.83–20.31, IQR = 6.11)	0.091	0.766 (0.563–1.044)
**Osteopontin [ng/mL]**	45.4 (0.19–423.94, IQR = 222.51)	13.24 (0.19–62.2, IQR = 37.42)	156.2 (0.24–423.94, IQR = 342.92)	**0.039**	0.960 (0.923–0.998)
**Leptin [ng/mL]**	0.9 (0.02–18.01, IQR = 2.63)	0.2 (0.03–1.85, IQR = 0.59)	2.7 (0.02–18.01, IQR = 5.37)	**0.013**	0.158 (0.037–0.677)

**Table 5:** Comparison of the concentrations of the assessed factors in patients with cystic tumors between their cystic fluid and serum in the cancer and non-cancer groups.

Variable	Group	Serum (Mean/Median)	Cystic Fluid (Mean/Median)	Mean/Median Difference (Serum-Fluid, SD/IQR)	*p*
**sHER-2 neu [ng/mL]**	Cancer	2.7 (0.08–8.66, IQR = 2.65)	2.7 (0.01–13.63, IQR = 3.99)	−0.6 (IQR = 3.36)	0.221
	Non-cancer	3.2 (1–6.7, IQR = 2.54)	5.3 (0.44–27.62, IQR = 12.87)	−2.2 (IQR = 10.03)	**0.026**
**sEGFR** **[ng/mL]**	Cancer	6 (0.32–26.21, IQR = 6.43)	2.6 (0.06–18.1, IQR = 4.71)	+2.8 (SD = 8.52)	0.229
	Non-cancer	8.1 (0.61–20.94, IQR = 7.31)	5.6 (0.74–33.67, IQR = 10.05)	−3.2 (SD = 11.22)	0.347
**sIL-6Ra** **[ng/mL]**	Cancer	7.8 (2.1–22.12, IQR = 4.32)	1.3 (0.01–6.36, IQR = 2.45)	+6.2 (IQR = 4.51)	**<0.001**
	Non-cancer	10.1 (3.66–25.42, IQR = 5.85)	4.4 (0.28–27.47, IQR = 15.09)	−3.6 (IQR = 13.84)	0.460
**Follistatin [ng/mL]**	Cancer	0.2 (0.001–0.67, IQR = 0.29)	0.3 (0.04–1.82, IQR = 0.5)	−0.2 (SD = 0.36)	0.087
	Non-cancer	0.3 (0.09–0.69, IQR = 0.25)	0.7 (0.06–1.55, IQR = 0.46)	−0.3 (SD = 0.36)	**0.005**
**FGF-basic** **[ng/mL]**	Cancer	0.1 (0.02–0.25, IQR = 0.08)	0.2 (0.05–0.91, IQR = 0.27)	−0.1 (IQR = 0.21)	**0.002**
	Non-cancer	0.1 (0.03–0.26, IQR = 0.08)	0.2 (0.1–0.89, IQR = 0.2)	−0.1 (IQR = 0.18)	**0.002**
**sVEGFR-2 [ng/mL]**	Cancer	0.7 (0.31–1.61, IQR = 0.33)	0.3 (0.01–1.2, IQR = 0.7)	+0.3 (IQR = 0.58)	**0.039**
	Non-cancer	1 (0.4–1.54, IQR = 0.38)	0.7 (0.1–4.98, IQR = 1.34)	+0.02 (IQR = 1.2)	0.955
**PECAM-1** **[ng/mL]**	Cancer	5.5(2–8.84, IQR = 1.9)	2.8 (0.15–7.58, IQR = 4.33)	+1.6 (SD = 2.26)	**0.011**
	Non-cancer	5.5 (3.39–8.51, IQR = 2.5)	4.5 (0.68–21.96, IQR = 8.23)	−2.1 (SD = 5.9)	0.186
**PDGF-AB/BB** **[ng/mL]**	Cancer	0.2 (0.001–1.79, IQR = 0.57)	0.2 (0.01–1.85, IQR = 0.51)	−0.1 (IQR = 0.57)	0.717
	Non-cancer	0.4 (0.013–4.54, IQR = 1.84)	0.1 (0.01–0.25, IQR = 0.18)	+0.3 (IQR = 2.18)	**0.039**
**Prolactin** **[ng/mL]**	Cancer	5.7 (0.01–108.67, IQR = 21.06)	0.7 (0.06–3.41, IQR = 1.37)	+17.5 (IQR = 24.66)	**0.001**
	Non-cancer	8.5 (0.01–143.8, IQR = 59.42)	1.2 (0.14–11.12, IQR = 3.22)	+5.8 (IQR = 40.53)	**0.019**
**G-SCF** **[ng/mL]**	Cancer	0.1 (0.003–0.25, IQR = 0.08)	0.1 (0.01–0.4, IQR = 0.14)	−0.02 (IQR = 0.18)	0.281
	Non-cancer	0.1 (0.08–0.18, IQR = 0.03)	0.1 (0.02–0.74, IQR = 0.2)	−0.02 (IQR = 0.11)	0.124
**HGF** **[ng/mL]**	Cancer	1 (0.21–2.24, IQR = 0.83)	3.29 (0.04–43.87, IQR = 8.53)	−2.6 (IQR = 8.1)	**0.011**
	Non-cancer	1.1 (0.3–1.96, IQR = 0.73)	16.6 (0.002–100.85, IQR = 78.32)	−17 (IQR = 82.16)	**0.002**
**sTIE-2 [ng/mL]**	Cancer	3.7 (0.16–17.01, IQR = 5.96)	0.9 (0.14–1.53, IQR = 0.74)	+0.9 (IQR = 3)	**0.008**
	Non-cancer	5.6 (0.42–11.43, IQR = 5.65)	1.6 (0.23–9.54, IQR = 3.05)	+3.2 (IQR = 4.78)	**0.023**
**SCF** **[ng/mL]**	Cancer	0.2 (0.002–0.38, IQR = 0.08)	0.1 (0.003–0.26, IQR = 0.13)	+0.1 (SD = 0.09)	**0.037**
	Non-cancer	0.1 (0.05–0.29, IQR = 0.13)	0.1 (0.02–0.6, IQR = 0.27)	−0.1 (SD = 0.14)	0.067
**sVEGFR-1** **[ng/mL]**	Cancer	0.0005 (0.0003–0.005, IQR = 0.003)	0.6 (0.001–7.42, IQR = 2.18)	−0.6 (IQR = 2.17)	**0.001**
	Non-cancer	0.0006 (0.0004–0.004, IQR = 0.001)	3 (0.83–20.31, IQR = 6.11)	−2.4 (IQR = 5.28)	**0.003**
**Osteopontin [ng/mL]**	Cancer	3 (0.1–17.85, IQR = 5.48)	13.24 (0.19–62.2, IQR = 37.42)	−12.9 (IQR = 32.87)	**0.005**
	Non-cancer	2.6 (0.06–6.1, IQR = 3.31)	156.2 (0.24–423.94, IQR = 342.92)	−179.5 (IQR = 354.38)	**0.001**
**Leptin [ng/mL]**	Cancer	1 (0.004–4.6, IQR = 1.17)	0.2 (0.03–1.85, IQR = 0.59)	+0.04 (IQR = 1.21)	0.279
	Non-cancer	2.1 (0.05–17.03, IQR = 5.76)	2.7 (0.02–18.01, IQR = 5.37)	−1.7 (IQR = 5.78)	**0.026**

## Data Availability

The raw data supporting the conclusions of this article will be made available by the authors upon request.

## Abbreviations

The following abbreviations are used in this manuscript:

FNA	fine needle aspiration
PDAC	pancreatic ductal adenocarcinoma
MCN	mucinous cystic neoplasm
SPN	solid papillary neoplasm
SCN	serous cystic neoplasm
IPMN	intraductal papillary mucinous neoplasm
IPMN-MD	IPMN main duct
IMPN-BD	IPMN branch duct
PD	pancreatoduodenectomy
DP	distal pancreatectomy
TP	total pancreatectomy
EUS	endoscopic ultrasound
CA 19-9 serum	carbohydrate antigen 19-9
WBC	white blood cell count
CRP	C-reactive protein
sEGFR	soluble epidermal growth factor receptor
sIL-6Ra	soluble interleukin-6 receptor alpha
FGF	fibroblast growth factor
sVEGFR-1/-2	soluble vascular endothelial growth factor receptor-1/-2
PECAM-1	platelet endothelial cell adhesion molecule-1
PDGF-AB/BB	platelet derived growth factor—AB/BB
G-CSF	granulocyte colony-stimulating factor
HGF	hepatocyte growth factor
sTIE-2	soluble TIE-2
SCF	stem cell factor
BMI	body mass index
ASA	American Society of Anesthesiologists
LEC	lymphoepithelial cyst
MRI	magnetic resonance imaging
CEA	carcinoembryonic antigen
IL	interleukin
IP-10	interferon gamma-induced protein-10
PDGFRβ	PDGF receptor β
PSCs	pancreatic stellate cells
Ang-1	angiopoietin-1
Ang-2	angiopoietin-2
